# Aberrant Hippocampal Subregion Networks Associated with the Classifications of aMCI Subjects: A Longitudinal Resting-State Study

**DOI:** 10.1371/journal.pone.0029288

**Published:** 2011-12-27

**Authors:** Feng Bai, Chunming Xie, David R. Watson, Yongmei Shi, Yonggui Yuan, Yi Wang, Chunxian Yue, Yuhuan Teng, Di Wu, Zhijun Zhang

**Affiliations:** 1 Medical School of Southeast University, Nanjing, China; 2 Department of Neurology, Affiliated ZhongDa Hospital of Southeast University, The Institute of Neuropsychiatry of Southeast University, Nanjing, China; 3 Department of Biophysics, Medical College of Wisconsin, Milwaukee, Wisconsin, United States of America; 4 Computational Neuroscience, University of Ulster, Derry, Northern Ireland, United Kingdom; Beijing Normal University, China

## Abstract

**Background:**

Altered hippocampal structure and function is a valuable indicator of possible conversion from amnestic type mild cognitive impairment (aMCI) to Alzheimer's disease (AD). However, little is known about the disrupted functional connectivity of hippocampus subregional networks in aMCI subjects.

**Methodology/Principal Findings:**

aMCI group-1 (n = 26) and controls group-1 (n = 18) underwent baseline and after approximately 20 months follow up resting-state fMRI scans. Integrity of distributed functional connectivity networks incorporating six hippocampal subregions (i.e. cornu ammonis, dentate gyrus and subicular complex, bilaterally) was then explored over time and comparisons made between groups. The ability of these extent longitudinal changes to separate unrelated groups of 30 subjects (aMCI-converters, n = 6; aMCI group-2, n = 12; controls group-2, n = 12) were further assessed. Six longitudinal hippocampus subregional functional connectivity networks showed similar changes in aMCI subjects over time, which were mainly associated with medial frontal gyrus, lateral temporal cortex, insula, posterior cingulate cortex (PCC) and cerebellum. However, the disconnection of hippocampal subregions and PCC may be a key factor of impaired episodic memory in aMCI, and the functional index of these longitudinal changes allowed well classifying independent samples of aMCI converters from non-converters (sensitivity was 83.3%, specificity was 83.3%) and controls (sensitivity was 83.3%, specificity was 91.7%).

**Conclusions/Significance:**

It demonstrated that the functional changes in resting-state hippocampus subregional networks could be an important and early indicator for dysfunction that may be particularly relevant to early stage changes and progression of aMCI subjects.

## Introduction

Mild cognitive impairment (MCI) is associated with a high risk for dementia [1], [2]. Amnestic type MCI (aMCI), which is characterized by the episodic memory loss, is a high rate of conversion to Alzheimer's disease (AD). The hippocampus, as part of the medial temporal lobe memory system and is one of the earliest brain regions affected by AD neuropathology, shows progressive degeneration as from aMCI progressed to AD [3].

Structural and functional MRI studies have consistently reported that altered hippocampus is a valuable predictor of conversion from MCI to AD [4]–[8]. Furthermore, the degree of hippocampal atrophy is significantly correlated with the impairment of memory and learning ability in AD patients [9], [10]. However, hippocampus is not an anatomically uniform structure but cytoarchitecturally, can be divided into different subregions such as cornu ammonis (CA), dentate gyrus (DG) and subicular complex (SUB) [11]. It receives diverse inputs which arrive via the entorhinal cortex, through the trisynaptic (signals from the entorhinal cortex to the DG, then to CA3, and finally to CA1) and the temporoammonic pathways (signals from the entorhinal cortex to CA1 and CA3), and its output travels via the SUB and fimbria/fornix [12], [13]. As the subregions of the hippocampus are spatially distinct, targeted regional measures of the hippocampal subregions are likely to provide more detailed information. Recent studies have revealed that subtle deficits of hippocampal subregion from structure MRI and task-related fMRI were observed in AD-spectrum subjects, i.e. deformations in CA and SUB [14], atrophy in CA and SUB [15], reduced cortical thickness in CA, DG and SUB [16], [17], hyperactivity [18] and hypoactivity [19] in CA and DG. The variance of aforementioned findings may due to the recruitment of different analytical methods, the characteristics of the task operations underway (i.e. complexity), and any differences between the regions in these studies may be related to difference in signal-to-noise ratio within the region of interest (ROI) (related to factors such as differences in voxel number in each ROI). However, these findings quite well highlight that the deficits of AD-spectrum subjects approximately seem to be associated with overall hippocampal subregions.

Resting-state fMRI, which provides a viable alternative imaging approach for the assessment of brain function in the low-frequency range (<0.1 Hz) of blood oxygenation level dependent (BOLD) fluctuations has also become popular in investigating brain changes in AD spectrum subjects. It has many advantages over other imaging methods, since application simply requires the participants to remain alert but relaxed during scanning; therefore, it has practical advantages in clinical applications, because no task engagement is required. In particular, functional connectivity measures temporal correlations of the spontaneous BOLD signals in different brain regions while subjects are at rest, therefore, could provide new insights into how structurally segregated and functionally specialized brain networks are interconnected. Although cross-sectional neuroimaging evidences have suggested that patients with AD could be characterized by abnormalities in resting-state functional connectivity of hippocampus [20]–[24], little is known about resting-state disturbances with the functional connectivity of specific hippocampal subregions in aMCI, and what values of these longitudinal changes would be of considerable interest.

The first purpose of the present study was to investigate the functional connectivity patterns of hippocampus subregional networks in aMCI subjects and controls using resting-state fMRI. Then, longitudinal changes in hippocampus subregional networks were further detected between aMCI subjects and controls at two time points separated by a mean period of 20 months. Finally, based on the different hippocampal subregions process information within an integrated and interconnected internal circuitry, we therefore derived longitudinal changes of these subregional networks to evaluate the contribution to clinical disturbances in these aMCI subjects and the values of classification in independent aMCI and control cohorts.

## Materials and Methods

### Participants

The study was approved by the Research Ethics Committee of Affiliated ZhongDa Hospital, Southeast University and written informed consent was obtained from all participants. It should be noted that the aMCI subjects had the capacity to consent because of no or minimal impairment in activities of daily living in the participans. The programs of participants' recruitment and follow-up information (i.e. dropouts) have been described in our previous study [25]. In the present study, aMCI group-1 (n = 26) and controls group-1 (n = 18) underwent the baseline and a mean follow-up period of 20 months (ranging from 15 months to 30 months) follow-up fMRI scans successfully. In addition, to compute the ability of these extent longitudinal changes (identified via aMCI group-1 and controls group-1) to separate subjects as well as to avoid circular analysis, we undertook classification analysis using three unrelated groups (i.e. these subjects were not included in aMCI group-1 and controls group-1) who only underwent the baseline fMRI scan, such as aMCI group-2 (n = 12), control group-2 (n = 12) and aMCI-converters who subsequently developed AD (n = 6).

### Subject testing

All subjects underwent diagnostic evaluations including a clinical interview and focused neurological and mental status exam, review of medical history, and demographic inventory. Cognitive functioning was evaluated by a mini mental state examination (MMSE) and the degree of dementia determined by a clinical dementia rating scale (CDR). In addition, a neuropsychological battery that consisted of Auditory Verbal Learning Test (AVLT)-delayed recall, Rey-Osterrieth Complex Figure Test, Digit Span Test, Symbol Digit Modalities Test, Trail Making Test-A, B and Clock Drawing Test to evaluate the function of episodic memory, attention, psychomotor speed, executive function and visuo-spatial skills respectively.

### Inclusion Criteria

Presence of aMCI, including single domain (the impairment involves only memory domain) and multiple domain (impairments in the memory domain plus at least one other cognitive domain), was made following the recommendations of Petersen [1] and others [26], including (1) subjective memory impairment corroborated by subject and an informant; (2) objective memory performances documented by an AVLT-delayed recall score less than or equal to 1.5 SD of age- and education-adjusted norms (cutoff of ≤4 correct responses on 12 items for ≥8 years of education); (3) MMSE score of 24 or higher; (4) CDR of 0.5; (5) no or minimal impairment in activities of daily living; (6) absence of dementia, or not sufficient to meet the NINCDS-ADRDA Alzheimer's Criteria (National Institute of Neurological and Communicative Disorders and Stroke and the Alzheimer's Disease and Related Disorders Association).

### Exclusion Criteria

Participants were excluded from the study if they had a history of known stroke, alcoholism, head injury, Parkinson's disease, epilepsy, major depression or other neurological or psychiatric illness, major medical illness, severe visual or hearing loss. Controls were required to have a CDR of 0, MMSE score ≥26, and an AVLT-delayed recall score >4 for those with 8 or more years of education.

### Longitudinal Follow-up

Follow-up neuropsychological tests and fMRI parameters were identical to those undertaken at baseline in every participant. Mean follow-up period was twenty months. Diagnostic and Statistical Manual of Mental Disorders-IV (DSM-IV) and NINCDS-ADRDA Alzheimer's Criteria were subsequently used to clinical diagnosis of AD.

### Magnetic resonance imaging procedures

The subjects were scanned using a General Electric 1.5 Tesla scanner (General Electric Medical Systems, USA) with a homogeneous birdcage head coil. Subjects lay supine with the head snugly fixed by a belt and foam pads to minimize head motion. Conventional axial Fast Relaxation Fast Spin Echo sequence T2 weighted anatomic MR images were obtained to rule out major white matter changes, cerebral infarction or other lesions: repetition time (TR) = 3500 ms; echo time (TE) = 103 ms; flip angle (FA) = 90^0^; acquisition matrix = 320×192; field of view (FOV) = 240 mm×240 mm; thickness = 6.0 mm; gap = 0 mm; no. of excitations (NEX) = 2.0. High-resolution T1-weighted axial images covering the whole brain were acquired using a 3D spoiled gradient echo sequence as follow: TR = 9.9 ms; TE = 2.1 ms; FA = 15^0^; acquisition matrix = 256×192; FOV = 240 mm×240 mm; thickness = 2.0 mm; gap = 0 mm. The functional scans (T2* weighted images) involved the acquisition of 30 contiguous axial slices using a GRE-EPI pulse sequence: TR = 3000 ms; TE = 40 ms; FA = 90^0^; acquisition matrix = 64×64; FOV = 240×240 mm; thickness = 4.0 mm; gap = 0 mm and 3.75×3.75 mm^2^ in-plane resolution parallel to the anterior commissure–posterior commissure line. This acquisition sequence generated 142 volumes in 7 min and 6 s. All subjects have eyes closed during scanning.

### Functional image preprocessing

Data analyses of groups were conducted with SPM5 (available at: http://www.fil.ion.ucl.ac.uk/spm). The first eight volumes of the scanning session were discarded to allow for T1 equilibration effects. The remaining images were corrected for timing differences and motion effects. Participants with head motion more than 3 mm maximum displacement in any direction of x, y, and z or 3 degree of any angular motion were excluded. The resulting images (both baseline and follow-up data) were spatially normalized into the SPM5 Montreal Neurological Institute echo-planar imaging template using the default settings and resampling to 3×3×3 mm^3^ voxels, and smoothed with a Gaussian kernel of 8×8×8 mm. Then, REST software [27] (available at: http://www.restingfmri.sourceforge.net) was used for removing the linear trend of time courses and for temporally band-pass filtering (0.01–0.08 Hz).

### Functional connectivity analyses

**Figure 1:**
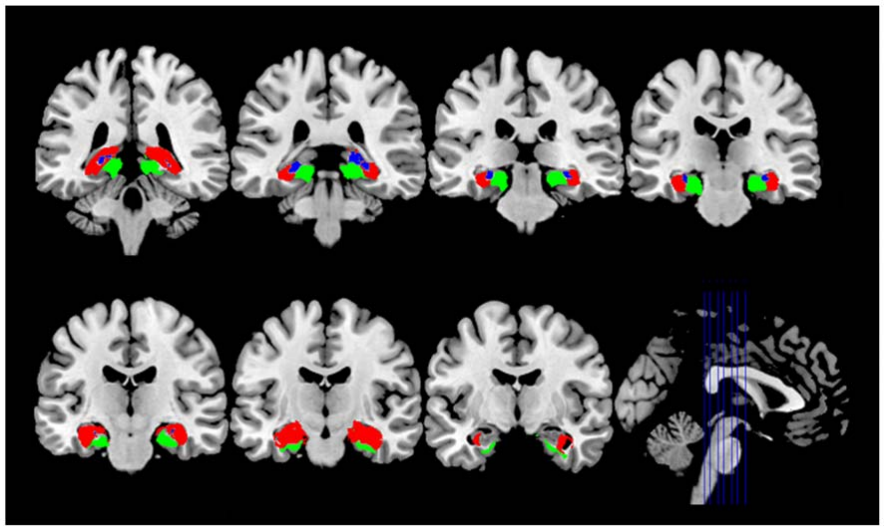
View of hippocampal subregions on coronal slices. Red: CA (CA1-CA3); Blue: DG (fascia dentate and CA4); Green: SUB (prosubiculum, subiculum proper, presubiculum and parasubiculum).

Seeds for the connectivity analysis corresponded to the six hippocampal subregions (CA, DG and SUB bilaterally, Figure 1), and were defined through the Anatomy Toolbox in SPM5 (available at: http://www.fz-juelich.de/inm/inm-1/DE/Forschung/_docs/SPMAnantomyToolbox/SPMAnantomyToolbox_node.html). For each subject, a mean time series for each hippocampal subregion was computed as the reference time course, separately. Cross-correlation analysis was then carried out between the mean signal change in each subregion and the time series of the voxels in the rest of the brain. A Fisher's z-transform was applied to improve the normality of the correlation coefficients [28]. Six head motion parameters and the mean time series of global, white matter and cerebrospinal fluid signals were introduced as covariates of no interest into each model. These analyses were performed using REST software.

### Voxelwise-based gray matter volume correction

To control for possible differences in hippocampal subregion networks that may be explained by differences in gray matter volume between subjects, we additionally performed an analysis of the probability of gray matter at a given voxel as a covariate (nuisance variable) in the analysis of voxel-wise and multi-subject resting-state functional data using standard statistical techniques [29]. The purpose of this method is presented to assess the component of the functional changes which cannot be attributed to anatomical difference and thus is likely due to functional difference alone. Firstly, Voxel-Based Morphometry (VBM5 toolbox, http://dbm.neuro.uni-jena.de/vbm) [30], [31] was used to explore modulated gray matter volume map of every subject. Secondly, these gray matter volume maps were transformed into the same final working space as the resting-state fMRI images using affine linear registration [32]. Thirdly, VBM results can be fairly sensitive to the size of the smoothing kernel used to smooth the tissue segment images. The criterion used in this work was to match the smoothness of the gray matter volume map data to that of the corresponding functional data (8 mm), in order to the spatial extent and magnitude of autocorrelation across the images. Finally, the resulting voxel-wise gray matter volume maps were as covariates in the analysis of functional data. The voxel-wise gray matter volume correction was recruited in all four groups. It noted that one subject of twenty-six baseline 3D structural data of aMCI group-1 had no anatomical images.

### Group-level analyses

Within group: to determine the patterns of functional connectivity of hippocampal subregions in each group-1, the spatial maps of functional connectivity in each group-1 were submitted to a random-effect analysis using one-sample t-tests. Statistical thresholds were set at a *P*<0.05 corrected by false discovery rate (FDR). To avoided potential interpretational confounds related to apparently negative connectivity resulting from correction for global signal changes [33], only positive functional connectivity was used in the present study.

Between groups: to explore whether longitudinal changes in the functional connectivity of hippocampal subregions were different in aMCI group-1 than in healthy controls group-1, a comparison between the change estimates between aMCI group-1 and controls group-1 using the contrast images (from baseline to follow-up), was conducted a voxelwise analysis of variance (ANOVA: group×hemisphere; networks of CA, DG and SUB were performed separately) at a *P*<0.05 corrected by FDR. It should be noted that ANOVA analyses were masked with a map from one-sample t tests of each relevant group, thresholded leniently at *P* = 0.05, uncorrected, and then combined across side and region to restrict connectivity analyses to positively correlated voxels only. This procedure avoided potential interpretational confounds related to apparently negative connectivity resulting from correction for global signal changes [33].

### Classification analysis

To avoid circular analysis, namely the use of the same data for selection and selective analysis will result in distorted descriptive statistics and invalid statistical inference whenever the test statistics are not inherently independent of the selection criteria under the null hypothesis [34]. Therefore, firstly, the overlap of the longitudinal changes in respective network of CA, DG and SUB identified via aMCI group-1 and controls group-1 was extracted as ROI. Secondly, we examined unrelated groups of baseline 30 subjects (aMCI group-2, n = 12; aMCI-converters who subsequently developed AD, n = 6; controls group-2, n = 12) and replicated the aforementioned analyses of hippocampus-subregion networks. Finally, the ability of ROI (mean Z values of overlap regions) to separate these subjects (aMCI group-2, aMCI-converters and controls group-2) was computed using Receiver Operating Characteristic (ROC) [35]. Area under the ROC curve (AUC) and best cutoff values were extracted, generating sensitivity and specificity values; the values distinguishing aMCI-converters from controls group-2, and aMCI-converters from aMCI group-2 were examined. The ROC was calculated using Medcalc software (available at: http://www.medcalc.org).

### Statistical analysis involving neuropsychological data

Nonparametric Mann–Whitney U-tests (MWU) were used for group comparisons of demographic and neuropsychological performance (statistical significance was set at *P*<0.05) at baseline and follow up using SPSS 15.0 software (available at: http://www.spss.com).

## Results

### Neuropsychological data

Healthy controls displayed levels of cognitive performance within the normal range both at baseline and follow up. Compared to controls group-1, aMCI group-1 showed mainly deficits in CDR, MMSE, and performance on AVLT-delayed recall and Rey-Osterrieth Complex Figure test-delayed recall (evaluate the function of episodic memory) both at baseline and follow up, confirming episodic memory impairment as a predominant symptom in these subjects (i.e. higher effect size than with other cognitive measures, Table 1). In addition, Table 2 showed the details of subject characterization in aMCI group-2, controls group-2 and aMCI-converters who subsequently developed AD.

**Table 1:** Demographic and neuropsychological data between aMCI group-1 and healthy controls group-1.

Items	Baseline				Follow up			
	aMCI	controls	*P*	Effect	aMCI	controls	*P*	Effect
	group-1	group-1	(MWU)	size	group-1	group-1	(MWU)	size
	(n = 26)	(n = 18)			(n = 26)	(n = 17)△		
Age (years)	71.4±4.3	70.3±4.7	0.357	-	-	-	-	-
Education levels (years)	13.8±2.8	15.1±3.1	0.084	-	-	-	-	-
Gender (male: female)	19: 7	10: 8	0.233	-	-	-	-	-
Clinical dementia rating	0.5	0	-	-	0.5	0	-	-
Mini mental state exam	27.2±1.5	28.3±1.3	0.026*	0.76	27.2±2.1	28.6±1.8	0.01*	0.69
Auditory verbal memory	2.8±1.2	8.1±1.9	0.000*	3.42	4.2±2.2	8.6±2.7	0.000*	1.79
test- delayed recall								
Rey-Osterrieth complex	32.9±4.7	34.7±1.4	0.471	0.47	34.0±2.9	35.0±0.8	0.715	0.42
figure test								
Rey-Osterrieth complex	12.0±7.4	17.3±6.8	0.017*	0.73	12.9±7.6	20.9±7.4	0.002*	1.04
figure test-delayed recall								
Trail making test-A (seconds)	88.7±36.2	70.0±28.7	0.049*	0.55	95.1±44.3	75.7±26.0	0.096	0.50
Trail making test-B (seconds)	182.3±70.4	139.3±39.2	0.046*	0.71	175.6±82.8	138.8±55.1	0.099	0.49
Symbol digit modalities test	27.7±10.4	34.3±8.7	0.043*	0.66	29.0±11.4	33.2±13.0	0.164	0.34
Clock drawing test	8.5±1.6	8.9±1.2	0.231	0.27	8.8±2.0	9.1±0.9	0.689	0.17
Digit span test	12.2±2.0	13.2±1.8	0.09	0.51	12.7±2.0	13.6±2.5	0.303	0.40

Note: Values are mean ± (SD); MWU: Mann-Whitney U-test, which was used here due to the neuropsychological data were not normally distributed;

*indicates had statistical difference between groups, *P*<0.05.

△ : One subject of follow-up neuropsychological data in healthy controls was absent. Effect size for distinguishing groups using Hedges g scores, accounting for sample sizes.

**Table 2:** Demographic and neuropsychological data in aMCI group-2, controls group-2 and aMCI-converters.

Items	Baseline	Baseline	Baseline	Follow up
	aMCI group-2	controls group-2	aMCI-converters	aMCI-converters
	(n = 12)	(n = 12)	(n = 6)	(n = 6)
Age (years)	72.3±5.7	71.5±6.1	72.7±4.9	-
Education levels (years)	13.2±3.8	13.6±3.1	13.3±3.0	-
Gender (male∶ female)	5∶ 7	6∶ 6	3∶3	-
Clinical dementia rating	0.5	0	0.5	1
Mini mental state exam	26.7±1.8	28.1±1.5	27.0±1.7	18.8±8.4
Auditory verbal memory test-delayed recall	3.0±0.7	7.7±2.0	1.5±1.8	0.3±0.5
Rey-Osterrieth complex figure test	31.6±4.7	32.5±4.2	19.3±15.7	17.6±19.3
Rey-Osterrieth complex figure test-delayed recall	11.9±7.9	14.3±7.7	4.8±6.5	0.3±0.8
Trail making test-A (seconds)	105.6±37.9	76.5±31.9	108.7±26.6	113.8±70.1
Trail making test-B (seconds)	189.8±82.7	140.1±54.0	237.8±92.8	225.3±135.6
Symbol digit modalities test	25.7±10.6	33.0±13.1	19.7±5.8	11.2±7.3
Clock drawing test	8.2±1.8	8.7±1.0	8.5±0.5	6.3±4.3
Digit span test	11.5±1.8	12.2±2.6	13.2±1.9	9.0±4.5

Note: Values are mean± (SD).

### Hippocampus subregional networks within-group analysis

**Figure 2:**
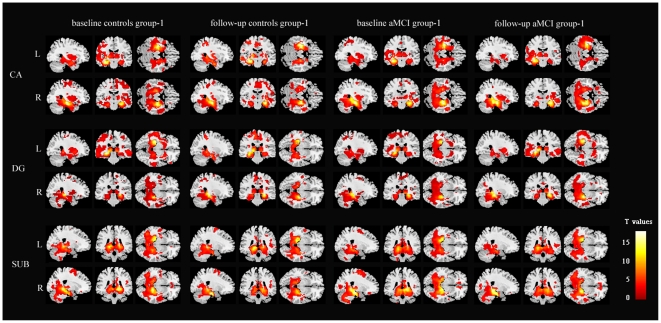
Validation of the networks of hippocampal subregions in aMCI group-1 and healthy controls group-1 at baseline and follow up. Distributed brain networks were demonstrated across the majority of clusters including diffuse subcortical, medial frontal, temporal cortical, parietal and cerebellar sites. Thresholds were set at *P*<0.05, corrected by FDR.

Both in aMCI group-1 and controls group-1 at baseline and follow up, each of six hippocampal subregions showed that the connectivity was stronger in areas close to the seed region. In addition, each hippocapus subregional network was composed of diffuse subcortical, medial frontal, temporal cortical, parietal and cerebellar sites (*p*<0.05 corrected by FDR, Figure 2). These functional connectivity patterns were similar to previous whole-hippocampus studies in terms of network connections [20]–[24].

### Longitudinal changes of hippocampus subregional networks between-group analysis

**Figure 3:**
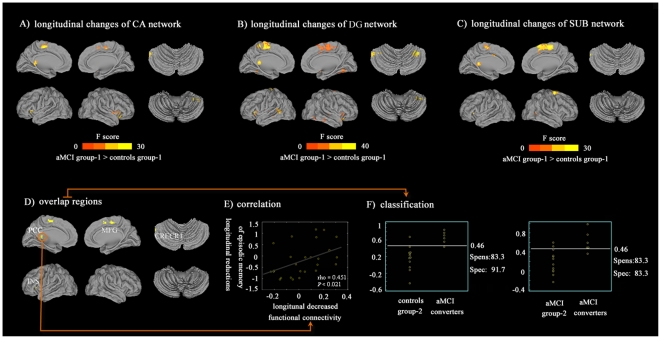
Longitudinal changes of hippocampus subregional networks. (A–C): Greater decreases in aMCI group-1 compared with controls group-1 between baseline and follow up. Thresholds were set at *P*<0.05, corrected by FDR. (D): Overlap regions from the conjunction analysis between the longitudinal changes of respective hippocampus subregional networks. Thresholds were set at *P*<0.05, corrected by FDR; PCC: posterior cingulate gyrus (left); MFG: medial frontal gyrus/supplementary motor area/paracentral lobule (bilateral); INS: insula (left); CRECR1: cerebellum_Crus1 (left); (E): Correlative analysis: within the aMCI group-1, the greater decreased functional connectivity between hippocampal regions and left PCC (i.e. average the Z values of the target in all networks) were positively related to greater reductions of episodic memory (AVLT-delayed recall scores, Spearman rho = 0.451, *p* = 0.021, two-tailed). It should be noted that the raw scores of AVLT-delayed recall for each subject was transformed to z scores. (F): Mean Z values of all sub-networks associated with overlap regions regarding the classification of independent groups: cutoff of 0.46 with sensitivity is 83.3%, specificity is 91.7%) in distinguishing aMCI-converters from controls group-2, while cutoff of 0.46 with sensitivity is 83.3%, specificity is 83.3%) in distinguishing aMCI-converters from aMCI group-2. It should be noted that these three groups only recruited the baseline data.

All the longitudinal changes of the networks of CA, DG and SUB, the connectivity patterns were bilateral and similar for seeds from the right and left hemisphere, and there were no significant connectivity clusters in the main effects of hemisphere and region×hemisphere ANOVA interaction analysis at a *p*<0.05 corrected by FDR. However, main effects of longitudinal changes of subnetworks showed that the differences between the aMCI group-1 and controls group-1 in terms of connectivity alteration over time were similar (*p*<0.05 corrected by FDR, Table 3 and Figure 3-A, B, C).

**Table 3:** Longitudinal changes of hippocampus-subregions networks in aMCI subjects group-1 (n = 26) compared to controls group-1 (n = 18).

Brain region	BA	Peak MNI	Peak	Cluster	Brain region	BA	Peak MNI	Peak	Cluster
		Coordiates	Z	size			Coordiates	Z	size
		x, y, z	score				x, y, z	score	
		(mm)					(mm)		
**(1) CA (Cornu ammonis, CA1-CA3)**					**(2) DG (Fascia dentata and CA4)**				
***Longitudinal changes: aMCI group-1>controls group-1***					***Longitudinal changes: aMCI group-1>controls group-1***				
L Insula/	13/	−30 21 0	4.49	621	L Insula/	13/	−30 21 0	3.89	891
Inferior Frontal Gyrus	47				Inferior Frontal Gyrus	47			
R Insula/	13/	33 21 −3	5.21	4941	R Insula/	13/	36 15 −12	6.11	3752
Inferior Frontal Gyrus	47				Inferior Frontal Gyrus	47			
L Medial Frontal Gyrus/	6	−3 −15 51	4.89	3024	B Medial Frontal Gyrus/	6	−3 −15 51	4.72	11610
Supplementary Motor Area/					Supplementary Motor Area/				
Paracentral lobule					Paracentral lobule				
R Medial Frontal Gyrus/	6	6 3 54	3.73	351	R Superior Frontal Gyrus	6	21 −6 75	3.83	783
Supplementary Motor Area/					L Superior Temporal Gyrus	41	−51 −18 9	3.54	621
Paracentral lobule					R Superior Temporal Gyrus	22	60 −24 0	3.73	1269
L Superior Temporal Gyrus	22	−48 −15 3	3.82	783	L Middle Temporal Gyrus	22	−54 −54 3	3.58	1458
L Posterior Cingulate cortex	29	−9 −45 3	3.95	702	L Middle Temporal_Pole	21	−42 15 −15	3.75	459
L Cerebellum_8/Crus1	-	−36 −48 −54	4.06	1728	R Middle Temporal_Pole	21	63 3 −18	4.24	459
***Longitudinal changes: aMCI group-1<controls group-1***					R Inferior Parietal Lobule	40	36 −48 42	3.27	297
none					L Posterior Cingulate cortex	29	−6 −42 9	4.05	702
					R Fusiform Gyrus/	18	27 −84 −9	3.71	891
					Lingual Gyrus	-			
**(3) SUB (Subicular complex)**					L Cerebelum_Crus1		−51 −51 −30	4.21	4374
***Longitudinal changes: aMCI group-1>controls group-1***					R Cerebelum_6	-	36 −48 −24	4.13	3213
L Insula	13	−33 24 0	4.66	567	***Longitudinal changes: aMCI group-1<controls group-1***				
B Medial Frontal Gyrus/	6	0 −21 57	4.74	15282	none				
Supplementary Motor Area/									
Paracentral lobule									
L Middle Cingulate gyrus	24	−6 0 36	3.96	567					
L Posterior Cingulate cortex	29	−6 −42 9	4.74	756					
R Middle Temporal Gyrus	37	48 −63 3	3.35	432					
L Cerebelum_Crus1	-	−54 −51 −36	4.06	270					
L Cerebelum_8	-	−27 −54 −57	3.65	324					
***Longitudinal changes: aMCI group-1<controls group-1***									
none									

Note: A corrected threshold of *P*<0.05 corrected by FDR was taken as meaning that there was a significantly difference between groups. BA: Brodmann area; MNI: Montreal Neurological Institute; R = right; L = left; B = bilateral; cluster size is in mm^3^.

Post hoc tests: Compared to controls group-1: (1) aMCI group-1 showed greater decreases in CA network integrity, including connections with the subcortex (bilateral insula), frontal cortex (bilateral medial frontal gyrus/supplementary motor area/paracentral lobule, bilateral inferior frontal gyrus), temporal cortex (left superior temporal gyrus), parietal cortex (left posterior cingulate gyrus) and cerebellum (left cerebellum_8/Crus1). (2) For DG, greater decreases in network strength were observed in connections with subcortex (bilateral insula), frontal cortex (bilateral medial frontal gyrus/supplementary motor area/paracentral lobule, bilateral inferior frontal gyrus, right superior frontal gyrus), temporal cortex (bilateral superior/middle temporal gyrus), parietal cortex (left posterior cingulate gyrus, right inferior parietal lobule), occipital cortex (right fusiform gyrus/lingual gyrus) and cerebellum (right cerebellum_6/left Crus1). (3) A comparative analysis of longitudinal changes for the SUB network displayed similar evidence of group related differences: subcortex (left insula), frontal cortex (bilateral medial frontal gyrus/supplementary motor area/paracentral lobule, left middle cingulate gyrus), temporal cortex (right middle temporal gyrus), parietal cortex (left posterior cingulate gyrus) and cerebellum (left cerebellum_8/Crus1). In addition, it should be noted that there was no evidence showed greater decreases in controls group-1 compared to aMCI group-1 in all hippocampus subregional networks.

### Overlap regions of differential hippocampus regional networks, behavioral significance and classification analysis

Conjunction analysis in longitudinal changes of respective subregional network found common regions with greater decreased connectivity in the left insula, bilateral medial frontal gyrus/supplementary motor area/paracentral lobule, left posterior cingulate gyrus (PCC) and left cerebellum_Crus1 (Figure 3-D). Then, we performed a correlative analysis between longitudinal changes in neuropsychological test scores and longitudinal changes of these overlap regions in all hippocampus subregional networks within aMCI group-1 and controls group-1. Within the aMCI group-1, the greater decreased functional connectivity between hippocampal regions and left PCC (i.e. average the Z values of the target in all networks) were positively related to greater reductions of episodic memory (auditory verbal memory test-delayed recall scores, Spearman rho = 0.451, *p* = 0.021, two-tailed) after controlling the effects of age, education, and gender (Figure 3-E). ROC analysis provided information on mean Z values of overlap regions regarding the classification of groups: AUC is 0.889 (*P*<0.0001, 95% Confidence Interval is 0.653 to 0.986, cutoff of 0.46 with sensitivity is 83.3%, specificity is 91.7%) in distinguishing aMCI-converters from controls group-2; AUC is 0.958 (*P*<0.0001, 95% Confidence Interval is 0.747 to 1.000, cutoff of 0.46 with sensitivity is 83.3%, specificity is 83.3%) in distinguishing aMCI-converters from aMCI group-2 (Figure 3-F).

## Discussion

The present study found that resting-state functional deficits were similar in six hippocampus subregional networks in aMCI subjects, and this impaired intrinsic connectivity was correlated with the decline of episodic memory. It also revealed that the functional index of these longitudinal changes allowed well classifying independent samples of aMCI and healthy controls, indicating their potential role as neuroimaging markers for monitoring the progression of aMCI that may in turn result in more rapid conversion to AD.

Altered function in the CA and DG regions represented a key component of early functional disturbances, which has been shown previous in task-related fMRI aMCI [18] and APOE-4 carriers [19] studies. However, the current study was the first time to localize key disturbances in the resting-state networks of hippocampal subregions (CA, DG and SUB) in aMCI subjects. While the networks detected for the subregions were approximately similar to previous whole-hippocampus studies [20]–[24], we also found very similarly longitudinal changes of respectively subregion-specific network in the aMCI subjects compared to controls over time. Three possible reasons as followings: firstly, more longitudinal deficits encompassing hippocampal areas (i.e. CA/DG/SUB network) may be a consequence of the spread of disease afterward; secondly, resting-state (i.e. unbiased by task demands) fMRI could detect any of these appeared to be particularly sensitive to change over time; thirdly, the resting-state function of hippocampus is associated with episodic memory processing [36], while the different hippocampal subregions process information within an integrated and interconnected internal circuitry: the main input into the hippocampal system is from entorhinal cortex, which is passed on for processing in CA and DG regions, finally the primary output to the neocortex leaves through the subiculum, fimbria/fornix, entorhinal cortex, and parahippocampal structures [12], [13], [37]. In addition, it is worth to mention that there was no evidence of connectivity decrements over time in the normal controls that were much more than aMCI subjects. Initially, this may suggest that any subregion-specific connectivity changes were not remarkably related to short-term normal aging effects but there were sensitive to the illness process.

The present correlative analysis revealed that the greater longitudinal deficits (hippocampal subregions-PCC) were associated with more impaired function of episodic memory (AVLT-delayed recall) in aMCI subjects, establishing their important role in progression of connectivity disturbances in aMCI. Episodic memory loss is the predominant symptom in aMCI subjects [1], [2], [26]. In addition, PCC has been considered as a tonically active region of the resting brain with high metabolic rates and an anatomic hub in the resting state brain [38]. Importantly, PCC also has been thought to be a valuable index of AD-related pathology, i.e. hypometabolism [39], hypoperfusion [40], amyloid deposition [41], volume reduction [42], reduction in regional homogeneity [43], activation [44] and functional connectivity [36], [39], [45] are associated with AD-spectrum subjects. Therefore, the present finding further suggested that altered cooperation of hippocampal subregions and PCC may be a key factor of clinical disturbances in aMCI.

A notable finding was that the significantly progressive deficits of functional connectivity of hippocampus regional networks could provide high classification accuracy of independent aMCI and control cohorts. Specifically, sensitivity and specificity of these regions were all more than 83.3%. Recently, several high dimensional classification methods have been proposed to automatically discriminate between patients with AD or MCI and elderly controls based on EEG [46], PET [47], T1-weighted MRI and CSF [48], suggesting signal of electrodes, morphometry, metabolism and CSF biomarkers were all sensitive to diagnostic status. However, in light of the present findings indicating also somewhat superior sensitivity and specificity of such resting-state fMRI measures, it does seem that these measures could be added in the candidates. In particular, these findings directly supported that hippocampus subregional networks may be valuable as neuroimaging markers for monitoring the development of these individuals who are with high risk of AD. The patterns of monitor is very important, as stable aMCI patients classified as having progressive impairment are likely to eventual conversion to AD and need to receive the most aggressive treatment.

There are, of course, technical and biological limitations in the present study. Firstly, there is a considerable clinical and biological heterogeneity in samples of present aMCI subjects whose recruitment were based only on clinical criteria. Some subjects may not display the underlying AD-pathology, and represent a ‘contamination’ of the sample with non-AD cases. This could be obtained by means of adding biomarker information to better characterize the study groups. Secondly, more subtly clinical typing (i.e. aMCI-single cognitive domain and aMCI-multiple cognitive domain) will be necessary to validate the finding. In addition, this study was performed in a relatively small cohort regarding to unrelated samples for classification, and replication of these findings in larger cohorts will be necessary for validation. However, the present findings explored the functional connectivity patterns of longitudinal changes in hippocampus subregional networks in aMCI subjects, which may have important clinical implications, as they suggested that the changes of functional connectivity in resting-state hippocampus subregional networks could be an important and early indicator for dysfunction that may be particularly relevant to early stage changes and progression of this disease.
